# Taking stock of an idiom’s background assumptions: an alternative relevance theoretic account

**DOI:** 10.3389/fpsyg.2023.1117847

**Published:** 2023-08-31

**Authors:** Ira A. Noveck, Nicholas Griffen, Diana Mazzarella

**Affiliations:** ^1^Laboratoire de Linguistique Formelle, CNRS and Université de Paris-Cité, Paris, France; ^2^Centre des Sciences Cognitives, Université de Neuchâtel, Neuchâtel, Switzerland

**Keywords:** idioms, idiom superiority effect, figurative language processing, theoretical approaches to figurative language, relevance theory

## Abstract

This paper begins by presenting the theoretical background of, and the accompanying psycholinguistic findings on, idiom processing. The paper then widens its lens by comparing the idiom processing literature to that of metaphor and irony. We do so partly to better understand the *idiom superiority effect*, according to which idiomatic sentences (unlike metaphoric and ironic ones) are generally processed *faster* than their literal controls; part of our motivation is to reconcile the differences between idiom processing, on the one hand, and metaphor and irony processing on the other. This ultimately leads us to Relevance Theory (RT), which has provided original insights into the processing of figurative language generally, but especially with respect to metaphor and irony. RT has paid less attention to idiomatic expressions (such as *break the ice*, *fan the flames*, or *spill the beans*), where one finds a single RT account that likens idioms to *conventional metaphors*. Through our overview, we ultimately arrive at an alternative RT account of idioms: We argue that idioms include a procedural meaning that takes into account relevant presuppositional information. For example, an idiomatic string such as *break the ice* not only asserts *initiate social contact*, it prompts the recovery of background assumptions such as *there exists a social distance that calls for relief*. This leads us (a) to apply linguistic-intuition tests of our presuppositional hypothesis, and; (b) to describe the paradigm and results from a pilot experiment. Both provide support for our claims. In doing so, we provide an original explanation for the *idiom superiority effect*.

## 1. Introduction

An idiom is a multi-word figurative expression whose constituent parts do not readily convey its intended meaning. For example, the literal meaning of the three words in the expression *break the ice* do not in themselves reveal its idiomatic meaning, which can be paraphrased as “initiate social contact.” While idioms may vary with respect to their opacity (consider how the words in *pop the question* more transparently reveal their figurative meaning, *viz.* “make a marriage proposal”), there is always a gap between an idiomatic expression’s literal and figurative meaning. This ostensible gap has been at the core of theory-making and experiments on idiom processing in the psycholinguistic literature since the late 1970’s, as it has been for other figures, such as metaphor, irony and metonymy (see Noveck, 2018).

The idiom processing literature has been shaped by two seminal accounts. On one side are those who argue that – despite appearances – there is nothing exceptional about idiom processing. According to the *compositional view* (Nunberg, 1978; Nunberg et al., 1994), idioms are processed word-by-word like any other expression. On the other side are those who argue that idioms call on processing that is distinctive from the sort needed to make a more literal reading. For example, according to the *direct retrieval view* (Bobrow and Bell, 1973), idioms exist in a separate, though parallel, lexicon of ordinary words. Once an independent lexicon is taken into account, one has to consider that there is an independent mode of processing. It was in the context of this debate that Swinney and Cutler (1979) defended their *lexical representation hypothesis*, which also assumed no special process for idioms; rather, these authors viewed idioms as akin to nominal compounds, such as *shrimpboat* or *hotdog.* This led the literature to consider other *hybrid views*, such as the *configuration hypothesis* (Cacciari and Tabossi, 1988), according to which people process an idiom compositionally until they recognize its idiomatic meaning, at which point it is retrieved in its entirety (Cacciari and Tabossi, 1988).^1^

Not surprisingly, this multiplicity of views has led to a large experimental literature that has provided several robust results. The best known of these is the *idiom superiority effect* which links idioms with a speed advantage when compared to non-idiomatic controls and across a wide range of tasks. Here, we mention four such cases. One comes from grammatical judgment tasks in which participants are asked to determine whether or not a sentence is a natural phrase (to appreciate a *rejectable* phrase, imagine foils such as *stranger is during*). In the context of such tasks, idiomatic expressions such as *spill the beans* (to mean *reveal a secret*) are evaluated as grammatical faster than their yoked controls, which for *spill the beans* would be something like *crate the beans* (see Swinney and Cutler, 1979). The second comes from the reading times of sentences in context. Ortony et al. (1978) reported that phrases intended to have an idiomatic reading were read more quickly than identical phrases whose context was designed to generate a literal meaning. For example, consider the idiom *let the cat out of the bag* (which also means *to reveal a secret*) when presented at the end of a vignette to describe the action of a protagonist who mistakenly revealed details of an upcoming surprise party; when presented in its control condition, this same string took longer to process when it was used to describe a saved kitten that emerged from its new owner’s paper bag (we will review this one in greater detail in the Section titled “A novel approach to idiom processing”). Third, consider a paraphrase task used in an experiment from Gibbs (1980), p. 150, in which participants were presented with an idiomatic phrase, such as *he’s singing a different tune*, under two conditions. In the figurative meaning condition, this phrase followed a story in which a politician changed his mind, making the phrase idiomatic; in the other, it followed a story in which a musician literally began singing a different melody. Participants were faster to endorse “he has now changed his mind” immediately after receiving the idiomatic version than they were to endorse “he’s not singing the same song” after they had been exposed to the literal one. Finally, one can see speed advantages for idioms in lexical decision tasks, in which probe words (which could be a real word or not) are presented immediately after an expression; unbeknownst to the participants, real words could be either related to the idiomatic or literal meaning of strings used in a brief sentence. Cacciari and Tabossi (1988) showed that probe words related to idiomatic meanings were more quickly recognized than those linked to literal readings (also see Tabossi et al., 2009; for a review, see Espinal and Mateu, 2019). The main take-home message of these sorts of findings is that idiomatic meanings are processed as quickly or faster than their literal controls. Phenomena linked to the *idiom superiority effect* continue to inspire investigations and to be a source of discussion (for recent work, see Canal et al., 2017; Carrol and Littlemore, 2020; Mancuso et al., 2020).

Interestingly, these data on idioms appear exceptional in the scope of figurative language processing in three ways. One is that, unlike the case for metaphor and irony, idiomatic processing has been investigated in the crucible of grammatical concerns (see Fraser, 1970 Nunberg, 1978; Nunberg et al., 1994). Not surprisingly, many studies on idiom processing focus on linguistic issues, such as syntax (Gibbs et al., 1989) or on semantic decomposability (Cutting and Bock, 1997). Early studies on idiom processing treated these expressions as ambiguous between literal and figurative meanings (Cacciari and Tabossi, 1988). Gricean theory or pragmatics were rarely mentioned in these discussions (for the exceptions, see Ortony et al., 1978; Gibbs, 1980). The second way that idiom-processing research stands out is that, from early on in this literature, idioms are often investigated in isolation; this means that there is a subset of studies that investigates idioms independently of context. This too is unlike the case for other well-researched figures. Metaphors have commonly been investigated as part of a full sentence, even if it is a short one (e.g., see Glucksberg et al.’s, 1982) *Some jobs are jails*] and are just as likely to be part of a longer vignette (e.g., see Ortony et al., 1978; Gibbs, 1991; Noveck et al., 2000; Almor et al., 2007). Ironic utterances are, practically by necessity, presented as part of a rich vignette (see Jorgensen et al., 1984; Pexman, 2008; Spotorno and Noveck, 2014). Finally, the effects from idiomatic processing, on the one hand, and metaphor and irony, on the other, are mirror images of each other. While idiomatic processing findings feature how idiomatic readings tend to be faster than their controls, the findings from metaphor and irony processing indicate that these figurative readings tend to be more cognitively demanding than their controls. One of the main aims of the current work is to reconcile the findings from the idiom processing literature with those in the other figurative language processing literatures. More specifically, we aim to revisit idiom processing from a more decidedly pragmatic perspective with the further aim of addressing the *idiom superiority effect*.

In the rest of this manuscript, we take the following four steps. First, we briefly review the processing literature on metaphor and irony in order to provide a fuller picture of figurative language processing more generally. Along the way, we will describe how Relevance Theory (the post-Gricean theory that is the unifying theme of the current research topic) has been impactful to the metaphor and irony literature and specifically by (a) underlining how intention reading is central to figurative language processing and (b) showing how each figure calls for its own detailed description. Second, we go on to consider the one Relevance Theoretic (RT) account of idioms that we know of (Vega-Moreno, 2001), which likens idiom comprehension to that of conventional metaphor. This section describes the added value that Vega-Moreno’s account brings to the literature but then considers how it does not anticipate the *idiom superiority effect*. Third, we develop our own original account of idiom processing. Like Vega-Moreno, we root our account in RT, but we argue that idioms share characteristics with presupposition in that (at least part of) their processing involves satisfying an idiom’s assumed preconditions. Once this assumption is made, one is in a better position to account for the *idiom superiority effect*. Finally, we present an armchair linguistic test that aims to test our account as well as an experimental paradigm that served as the basis for a pilot study. The conclusions from both tests provide support for our account.

## 2. Figurative language processing: looking under the hood

### 2.1. A focus on metaphor and irony processing

The basis for many of the early studies on figurative language was inspired by Gricean Theory, which, as Gibbs and Colston (2012, p. 65) wrote, “assumed that people must always do a complete literal analysis of an expression before any pragmatic information is evaluated to derive speaker meaning, which in turn implies that figurative language processing must always take longer than literal speech comprehension.” As data accrued on figurative language processing, it became clear that this need not be the case. At some point, Grice’s so-called Standard Pragmatic Model (fairly or unfairly) became the reference that experimental post-Gricean accounts typically argued against (for a discussion, see Noveck, 2018). The upshot is that several new accounts emerged, two of which played a large part in forming the psycholinguistic literature on figurative language processing.^2^ One is Gibbs’s (1986) and Gibbs et al.’s (1989) *Direct Access* view, which claims that the non-literal meaning of a given word or phrase is accessed without considering its literal meaning. However, as even more data were collected, much of them showing that metaphoric and ironic meanings often *are* associated with slowdowns compared to literal readings, it was hard to maintain this argument [see (Gibbs and Colston, 2012), for arguments *against* the Direct Access view]. The other important position to emerge on figurative processing was championed by Giora (1997), who argued that conventional and frequent (i.e., the most salient) meanings of words are processed first. Thus, slowdowns related to figurative readings are likely due to those situations in which participants consider literal meanings first. However, according to Giora’s *Graded Salience* view, literal meanings *can be* superseded by accessing a word’s figurative meaning (with respect to irony, see Giora et al., 1998, 2007; Giora and Fein, 1999; Schwoebel et al., 2000).

As this theoretical introduction indicates, one of the main dependent measures in the study of metaphoric and ironic processing is the relative reading time speeds comparing a figurative reading of a sentence to a baseline. If one were to find evidence indicating that the understanding of a metaphoric or an ironic sentence, say, generates longer latencies than its literal reading, one could argue that figurative processing is effortful; if one does not find differences (i.e., null results), one could argue that there is nothing unique about figurative language. The juxtaposition of these two kinds of possible findings characterizes exchanges and debates in the psycholinguistic literature since its beginnings with respect to ironic and metaphoric materials. It would be fair to say that, overall, one finds that figurative readings *can* indeed be accessed as fast as literal ones; however, *ceteris paribus* and with minimal context, figurative language processing is generally associated with slowdowns.

To appreciate the kind of data that these theories aimed to account for, one can hark back to some of the earliest studies on metaphor processing. For example, Ortony et al. (1978) prepared vignettes in which a test sentence could be placed in one of two contexts, one that would render the test sentence metaphorical and the other literal. Consider the test sentence, *Regardless of the danger, the troops marched on* when it is preceded by just one line of text. In order to elicit a metaphoric reading, that preceding line concerned children who were annoying their babysitter; in order to elicit a literal reading, the authors provided a line concerning soldiers in battle. With such limited contexts, the test sentence was read significantly more slowly in the metaphoric condition than in the literal condition. However, when the preceding text was expanded to a paragraph (thus providing much more referential detail), the latencies of figurative versus literal readings of critical test sentences were dramatically reduced (for another early study in the same direction with short sentences and ERPs as dependent measures, see Pynte et al., 1996). For metaphor (as well as for irony), it is generally the case that figurative meanings take longer to process than their literal controls, and especially when there is little background information. However, contextual or experimental features can indeed reduce these differences, to the point that figurative cases can appear as fast as their literal controls. Note, again, that the *idiom superiority effect* makes a different stronger claim, which is that idiomatic readings are processed *faster* than their literal controls.

### 2.2. Relevance theoretic approaches to irony and metaphor

Relevance Theory has played a prominent role in accounting for irony and metaphor. According to Sperber and Wilson (1986/1995) determining the meaning of an utterance is part of a listener’s effort to understand the speaker’s intended meaning which is always inferred (even when it consists in deriving a literal interpretation of an utterance). The inferences involved, however, make the comprehension of an utterance vary with respect to the effort they require. Both the sentence meaning and its context contribute to making some interpretations more easily derivable than others. For both irony and metaphor, it is not a single application of a general rule (such as *Direct Access* or *Graded Salience*) that generally determines how figurative language is processed and whether a figurative meaning requires more (or less) effort to process than its literal use. Rather, comprehension difficulty depends on the intention that the speaker wants to communicate and the inference-making that it takes to read that intention. While there were Relevance Theorists who carried out experiments from early on (on irony, see Jorgensen et al., 1984), nearly all of their arguments about figurative language were theoretical until the turn of the century when experimental studies inspired by Relevance Theory became more commonplace (see Noveck et al., 2000; Noveck and Sperber, 2007).

As far as irony is concerned, RT emphasizes the *echoic* use of language, in which comprehension depends on detecting that the speaker wants to convey a skeptical, mocking or dissociative attitude about a previous thought. For example, imagine two professional opera performers who unexpectedly sing horribly (see Spotorno and Noveck, 2014). When one singer says (1) ironically to the other, she is mocking herself and her colleague for having had more lofty expectations.

(1) Tonight we gave a superb performance.

Spotorno and Noveck (2014) showed that ironic readings of lines of text like those in (1) do indeed take longer to process compared to literal readings of the same lines (to appreciate [1] literally, imagine a context in which the vignette describes the singers as having performed well before the critical test sentence is presented). However, the authors also reported *Early-Late* (trial) *effects*, i.e., differences in reading time speeds between the two conditions largely disappear by the time a participant comes to the end of their experimental session (particularly when ironic utterances in a reading task arise consistently after a negative event). Their explanation was that it is intention reading (or Theory of Mind) that intervenes as the speaker’s – or perhaps the narrator’s – intention becomes more obvious as the number of ironic reactions populate the experimental session (for a recent extension on such intention-reading claims, see Ronderos et al., 2023). Incidentally, neuro-imaging studies show that participants’ irony-processing appears to rely on brain regions highly associated with Theory of Mind processes (Spotorno et al., 2012). In short, for the RT approach, it would not be surprising to find that ironic readings could be carried out with a speed that is comparable to literal readings as long as the ironic line is consistent with the speaker’s apparent intention.

In contrast, the standard RT account views metaphor as a form of ‘loose use of language’ comparable to other phenomena, such as hyperbole and neologism, in which the meaning communicated by the use of a word in context ultimately differs from the linguistically encoded meaning of that word. Through a general pragmatic process known as ‘concept adjustment’ (Carston, 2002; Wilson, 2003), a word could convey a more specific concept, as the word *drink* does in *Let me buy you a drink* when heard at a bar to mean “an alcoholic drink.” Or, it could convey a concept that is more general than the lexical concept. For example, the shape in *France is hexagonal* is very much a loose use of that mathematical object. Metaphors, according to RT, involve a combination of narrowing and broadening (Carston, 2002) that helps guide the listener to understand the concepts that the speaker intends to communicate.

Rubio-Fernandez (2007) investigated metaphor in a Relevance framework through a cross-modal priming study. She argued that the enhancement of the relevant properties of the metaphor, and suppression of those that are irrelevant for the figurative interpretation, is a necessary process in metaphor comprehension. In order to test her claim, she presented 20 two-sentence-long vignettes whose second sentence would often conclude with a metaphor. For example, participants would read sentences such as (2):

(2) Nobody wanted to run against John at school. John was a cheetah.

By presenting probe words immediately after the metaphor (i.e., zero seconds after the final word) or else at 400 ms or 1,000 afterward, she was able to profile the way metaphoric meanings emerge. That is, she presented three different kinds of probe words – (i) an unrelated term (e.g., *plant*), (ii) a superordinate, literally related, term (*cat*) or; (iii) a distinctive, figuratively related, term (*fast*) – after items like those in (2) and reported three findings. First, the *immediate* reactions to the three types of probes revealed that the unrelated meaning is significantly slower than both the metaphor’s literal superordinate meaning *and* the metaphor’s intended meaning. Second, listeners continued to show a speed advantage for the superordinate probe over the unrelated one at 400 ms but this preference disappeared at 1,000 ms. Third, like the superordinate probe, the distinctive-property probe also had a speed advantage over the unrelated one at 400 ms; unlike the superordinate word, the distinctive probe maintained its advantage over the unrelated one at 1000 ms. She concluded that metaphor interpretation involves enhancing relevant properties of the metaphor vehicle while actively *suppressing* the superordinate associations.

## 3. A relevance theoretic approach to idioms: are they akin to conventional metaphors?

The question of how idioms are processed has received less attention from relevance theorists as compared to other figurative uses of language. One notable exception is the work from Rosa Vega-Moreno (2001, 2003, 2005), whose relevance-theoretic account views idiom comprehension as comparable to other loose uses of language. Her account underlines the importance of thinking of idiomatic expressions as essentially irreducible to their literal paraphrase; as she puts it (Vega-Moreno, 2001, p. 76): “idioms cannot be paraphrased without loss.” For instance, she argues that the idiom *kick the bucket* cannot be aptly paraphrased with the verb *die*. Although the two encode conceptual representations that are logically related (anybody who kicked the bucket died), the mental representation associated with the idiomatic expression contains additional information about the manner of death (people who kicked the bucket presumably died suddenly and unexpectedly), the attitude that one has (for this example, we assume she means the attitude toward the deceased) as well as “something imagistic” (Vega-Moreno, 2001; p. 76), all of which are not associated with the concept encoded by the verb *die*. As a result, far from being a rhetorical device, the use of an idiomatic expression would be motivated by the speaker’s intention to convey a meaning that could not be conveyed otherwise. But how is this meaning inferred by the hearer?

Vega-Moreno (2003, 2005) suggests that the interpretation of an idiomatic expression relies on a pragmatic inferential process that can take as its starting point the meaning encoded by the idiom string (a holistic and structured conceptual representation), as well as the lexical meaning of its constituents. When processing the idiomatic string, both the structured phrasal concept encoded by the idiom and the concepts lexically encoded by the individual constituents are activated. As with metaphors or other loose uses of language, these meanings are subject to a process of conceptual adjustment, which allows the construction of *ad hoc* concepts. As a result, they can convey an occasion-specific meaning related to the particular use of the word or phrase at issue and can ground the derivation of relevant implications (see Wilson and Carston, 2007).

According to Vega-Moreno (2003), the interpretation of idiomatic expressions typically involves the interplay between the pragmatic adjustment of the concepts lexically encoded by the idiom’s constituents as well the construction of an *ad hoc* phrasal concept. To illustrate this, consider her main example:

(3) I cannot stand the way my boyfriend is tied to his mother’s apron strings.

To interpret the expression “tied to his mother’s apron strings,” a listener could start with the encoded concept TIE and pragmatically broaden it to include in its denotation any process in which some degree of attachment is involved (thus constructing an *ad hoc* concept TIE*). Crucially, though, this on-line conceptual adjustment would be complemented by accessing the meaning of the idiomatic expression as a whole:

At some point during this process, the concept encoded by the idiom string as a whole is retrieved from memory ([TO BE TIED [TO [ONE’S MOTHER’S APRON STRINGS]]]*). Rather than involving a switch of processing mode, the hearer takes this concept also as a further clue to the speaker’s meaning and he starts considering some of its accompanying information (e.g. the assumption that someone with this property is too close to their mother, not independent enough for their age, and so on) (Vega-Moreno, 2003, p. 313)

As a result, idiom comprehension would involve a process of conceptual adjustment of the meaning of the individual constituents (which typically involves broadening) as well as a process of conceptual adjustment of the stable conceptual representation associated with the idiomatic string in memory (which typically involves narrowing). Indeed, because the conceptual representation that is associated with the idiom string in memory is often unspecified, it regularly requires some pragmatic specification.

Crucially, the relative role of conceptual adjustment with respect to the meaning of individual constituents depends on the degree of decomposability and transparency of the idiomatic expression; that is, each constituent contributes independently and in an identifiable way to the idiomatic interpretation. In the case of decomposable idioms, the greater the idiom transparency, the greater the contribution of the pragmatic adjustment of the meaning of individual constituents. In the case of non-decomposable idioms (e.g., *chew the fat*), the meaning of the constituent words does not contribute at all to the recovery of the idiom meaning, so any process of conceptual adjustment at a word level may disrupt, rather than contribute to, the derivation of the idiomatic interpretation. Although consistent with some available empirical data suggesting that the understanding of compositional idioms is often facilitated as compared to non-compositional strings (Gibbs, 1991), note that this account does not address the *idiom superiority effect*. At least it is not clear what would make this explanation account for faster idiomatic reading times when compared to its controls.

Vega-Moreno’s account displays, however, some interesting features, which we incorporate in our analysis of idiom comprehension. First, it acknowledges the richness of the meaning that is conveyed by idiomatic expressions and its irreducibility to a literal paraphrase. Second, it emphasizes the pragmatic dimension of idiom understanding: far from being a matter of pure linguistic decoding, the processing of idioms involves a great deal of pragmatic inference to recover the speaker’s intended meaning. Indeed, in line with the relevance-theoretic framework, Vega-Moreno (2003) conceives conceptual adjustments at the word or phrasal levels as part and parcel of the search for a relevant interpretation of the speaker’s utterance containing the idiom. As a result, idiom comprehension relies on pragmatic enrichments typically involving broadening of the lexically encoded meaning of individual constituents as well as narrowing of the conceptual representation associated with the idiomatic string as a whole. This involves “a simultaneous adjustment of word, phrase and sentence meaning which take[s] place during the process of deriving the explicit content, *context* and implicatures” (Vega-Moreno, 2003, p. 312, our emphasis).

In what follows we take these points a step further and suggest that a full-fledged account of idioms requires spelling out how the idiomatic interpretation contributes to the derivation of the appropriate context. We argue that understanding an idiomatic expression involves the derivation of a series of background contextual assumptions, whose use is critical in order for the idiom to be understood.

## 4. A novel approach to idiom processing

### 4.1. The background

The genesis of the account that we are about to present emerged while reviewing experimental papers on idiom processing in the context of the figurative language processing literature generally. It was observed that – typically – when an idiomatic expression was employed as part of a vignette in a behavioral task, the figurative meaning was sensible because the context contained several elements that justified its use, i.e., it was felicitous in context. For example, consider the item below (4a) from one of the early studies on idiom comprehension (Ortony et al., 1978):

(4a) Dean spoiled the surprise that Joan had been planning for their mother’s birthday party. When he realized what he’d done, he apologized for having let the cat out of the bag.

One can see that the reader is informed that there was a surprise that was ruined so the expression *letting the cat out of the bag* fits with a prior situation. In contrast, when an idiomatic phrase is used to convey a literal meaning, it would naturally make the idiomatic meaning nonsensical in context, if one were to assume that the figurative meaning was indeed generated. For example, consider the literal control (4b) for the vignette above:

(4b) Walking back from the store, Anne found a kitten which she put in with her groceries. She got home and her puppy went wild when she let the cat out of the bag.

In this case, *letting the cat out of the bag* is designed to be understood literally. Note though that – if the idiomatic meaning were to be generated – it would come without any contextual support. There is no secret that was betrayed so the idiomatic reading would be incongruous with the previous information.

This appears to be a general feature of idiom studies that use vignettes: when a string is employed and intended to be understood idiomatically, its specific preconditions were met in the prior context and when the literal controls of idioms are employed, (a) the investigated strings are assumed to be stripped of their idiomatic meaning and; (b) the vignettes are naturally presented without the contextual support that would make the string’s idiomatic meaning felicitous [for other such examples, see (Gibbs, 1980)]. With this insight in tow, one has the beginnings of a sensible explanation for the *idiom superiority effect*. That is, it indicates that the well-known effect is not necessarily due to idiom processing being in some way accelerated; it is arguably due to the fact that the literal control items (which still use idiomatic strings) prompt slowdowns because they (a) likely generate figurative meanings which are then (b) incongruous with the context.

The upshot worth noticing is that the felicitous use of idiomatic expressions appears to require some contextual preconditions (which vary from idiom to idiom) that are needed to make the idioms apt. For instance, the idiomatic meaning of *break the ice* is felicitous in contexts characterized by an initial tension between strangers (see Levorato et al., 2004), while *fan the flames* is sensible in contexts where there is a pre-existing and ongoing conflict and *spill the beans* is reasonable in contexts in which there is a secret to reveal, and so on and so forth. We take this observation as a starting point to make a fundamental suggestion: that idioms are accompanied by a set of background assumptions, which verge on being presuppositional. To develop this proposal, we first clarify the notion of presupposition that we have in mind (section “Presuppositions”) and then elaborate on the way in which idiomatic expressions can also be conceived as carrying presuppositional-like effects (section “Idioms and presupposition-like effects”).

### 4.2. Presuppositions

The idea that information can be presupposed, as opposed to being explicitly asserted, has a long history in philosophy of language and linguistics (for an overview, see Beaver et al., 2021). Presuppositions are standardly described as backgrounded information, that is, information that is old, given or taken for granted by the interlocutors (or at least presented as such). For the purpose of this paper, we focus on the way in which this notion has been integrated into Relevance Theory. In her seminal work, Simons (2005) describes presuppositions as “relevance requirements” or “relevance establishers”; that is, they are background assumptions that contribute to the relevance of the overall interpretation by giving access to a context in which further assumptions (the explicatures or implicatures of the utterance) can achieve contextual effects (see also de Saussure, 2013; Mazzarella and Domaneschi, 2018). According to Simons, presuppositions are thus “the propositions which that addressee must accept for the utterance to be relevant in the way intended by the speaker” (Simons, 2005, p. 333). For instance, to achieve relevance in the way intended by the speaker, an utterance, such as *Even Trump admitted that climate change is real* (adapted for our purposes from Simons) requires the background assumption that Trump is a particularly unlikely person to make such an admission, based on his previously shared views on the matter. The addressee needs to accept this presupposition to infer some intended implications, such that the evidence of rapid climate change is undeniable, that this should be a concern for the environmental policy of all parties, etc.

The linguistic literature on presupposition has identified a variety of lexical items or constructions that trigger the derivation of presuppositions (and are thus known as “presupposition triggers,” see Karttunen, 1974; Levinson, 1983). These include expressions such as factive verbs or change-of-state verbs, which trigger presuppositions that are undetachable from what is said. For instance, by uttering “Deirdre *left* the house,” the speaker presupposes that she was in the house immediately before the reference time and asserts that she is no longer in the house (where the asserted content cannot be expressed without triggering the presupposition). Furthermore, the class of presupposition triggers also include lexical items such as *again*, *too*, *even*, whose only function seems to be the triggering of the presupposition itself. By uttering “Deirdre laughed *again*,” the speaker is using *again* to presuppose that she laughed before (for a discussion on the distinctive features of these “dedicated presupposition triggers,” see Simons, 2005). Drawing on the relevance-theoretic notion of “procedural meaning” (Blakemore, 1987, 2002; Simons, 2005) suggests that presupposition triggers encode dedicated procedures, which guide the inferential comprehension process by imposing constraints on the construction of contexts. These expressions can thus “indicate that the speaker intends (the truth conditional content of) her utterance to be interpreted relative to a context which contains ‘the presupposition’” (2005, p. 349).

The role of context has been widely investigated with respect to the processing of statements containing presupposition triggers. Indeed, many experimental studies have explored the contrast between situations in which a presupposition is contextually supported, or “satisfied,” and a situation characterized by a “presupposition failure” which cannot be readily repaired (e.g., see Ferretti et al., 2008, 2013; Jouravlev et al., 2016; Shetreet et al., 2019; for a review, see Schwarz, 2015 or Reinecke, 2020; for formal distinctions, see Glanzberg, 2003). In the former case, the linguistic context already includes or entails the background assumption that is linguistically triggered by the presuppositional statement. In the latter case, not only the presupposed content is unavailable (not taken for granted by the interlocutors), but it is also inconsistent with the immediately preceding linguistic context, thus making it impossible for the hearer to accept it (or “accommodate” it, see Lewis, 1979). Consider a straightforward example from Jouravlev et al. (2016), who compared two kinds of stimuli (e.g., 5a and 5b) as part of an EEG study:

(5a) Jake had tipped a maid at the hotel once before. Today he tipped a maid at the hotel again.

(5b) Jake had never tipped a maid at the hotel before. Today he tipped a maid at the hotel again.

These authors reported late widely distributed positivity after the onset of the trigger *again* (indicative of an early arriving P600 effect) when the presuppositional trigger was inconsistent with the previous content (in 5b) as opposed to consistent with the previously stated context (in 5a). This indicates that presupposed information is processed differently depending on whether the context is supportive or not. Not surprisingly, presuppositional content is integrated more smoothly when it is consistent with preceding information.

### 4.3. Idioms and presupposition-like effects

We want to suggest that idioms are also typically accompanied by a set of background assumptions, making these figures verge on being presuppositional; i.e., the relevance of an utterance containing the idiomatic expression depends on the recovery of these assumptions. For instance, by using the idiom *break the ice* in “Joey broke the ice by making a joke,” the speaker appears to presuppose a range of background assumptions that includes the presence of an initial tension among a relevant group of people, a tension which is palpable and which nobody had yet tried to mitigate. We adopt Simons’s (2005) language to describe these background assumptions as presuppositional. That is, they are meant to contribute to the relevance of the overall interpretation by setting up a context in which the claim “Joey broke the ice by making a joke” can be interpreted as implying that Joey was motivated to change this uncomfortable interpersonal dynamic (thus functioning as “relevance establishers”). Similarly, the idiom *fan the flames* in “She fanned the flames” appears to presuppose the existence of preexisting tension, characterized by a certain degree of animosity among the people involved. Recovering these background assumptions plays a crucial role in constructing the context in which “She fanned the flames” can be interpreted as suggesting that she acted in a way that is likely to feed this existing conflict and aggravate it, to worsen the personal relationship at stake, etc. Similar considerations can be applied to a variety of idioms (*make a killing*, *hit a wall*, *clip one’s wings*, *spill the beans*, etc.), thus indicating that processing an idiom routinely involves accessing a variety of background assumptions that shape the context of interpretation.

How are these background assumptions brought about in the interpretation process? In the literature on presuppositions, we find a well-established distinction between the so-called “pragmatic presupposition” and “semantic presupposition” (see, e.g., Potts, 2015). While the former is entirely pragmatically motivated (see also de Saussure, 2013 on “discursive presupposition”), the latter traces to conventional aspects of the meanings of specific words and constructions, the class of presupposition triggers discussed above. The status of the background assumptions associated with an idiomatic expression is far from being settled, and it may well differ from idiom to idiom. In what follows, though, we explore our original hypothesis, which is that idioms encode procedural meanings that work as instructions for the recovery of these assumptions.

According to our hypothesis, idioms encode not only a conceptual component but also a procedural one. Following Simons’ account of presupposition triggers as procedural meanings, idioms are thought of as encoding procedures to construct the relevant context of interpretation, one which includes the set of background assumptions which make the use of the idiom felicitous. For instance, the idiomatic expression *break the ice* would thus encode procedural indications to recover assumptions related to the presence of palpable tension among the people at issue. As suggested by Wilson (2011, p. 9), “[t]o say that a word encodes a certain concept or procedure is to say that the linguistic system is linked to the rest of the cognitive system in such a way that activating the word will systematically activate the associated concept or procedure.” It follows from this that if idioms encode both a phrasal concept and a procedure, processing the idiomatic string will systematically result in activating both, thus triggering a process of inferential reconstruction of a set of relevant background assumptions.

In line with standard examples of presupposition triggers, most idioms would typically prompt the recovery of a precise and identifiable set of background assumptions. It is also worth noticing that certain idioms additionally invite the recovery of broader (and vaguer) arrays of assumptions, attitudinal dispositions or imagistic components, that could be described in terms of so-called “non-propositional effects” (Wilson and Carston, 2019). Consider, for instance, how *kick the bucket* conveys a specific attitude toward the deceased. That is, reconsider Vega-Moreno’s (2003) example for *kick the bucket* in (6):

(6) Has horrible old Mr. Thomas kicked the bucket yet?

Clearly, the dissociated, distant, or even negative attitude toward the referent of this idiom (*horrible old Mr. Thomas*) plays a role in comprehending the idiom. To appreciate the role of attitude, compare (6) to *Has the love of my life, my inspiration, Tom, kicked the bucket yet?* In this latter case, the choice of idiom, taken at its face, appears to be incongruous.

We suggest that the hypothesis that idioms encode procedural meanings meshes well with these observations. Interestingly for our purposes, the notion of procedural meaning has also been employed to capture the expressive dimension of a range of communicative devices – interjections, emotional prosody, expletives, etc. – which are regularly associated with the expression of an emotive attitude (see, e.g., Wharton, 2003; Wilson and Wharton, 2006; Blakemore, 2011). In all these cases, the encoded procedures are taken to activate representations of emotional states, evaluative contents or attitudinal descriptions (for a discussion, see Carston, 2016). The notion of procedural meaning thus seems to be well suited to account for the presuppositional-like effects of idiomatic expressions, even when these pertain to less determinate and more nuanced contents.

Finally, this hypothesis can shed additional light on the claim that idioms are irreducible to their literal paraphrase, so that - as discussed before – *kick the bucket* cannot be paraphrased as *die* without loss (Vega-Moreno, 2001). One possibility is to think of the meaning that is lost in the paraphrase as linked to the procedural meaning idioms encode. It is arguably the procedural meaning encoded by the idiom that allows for the richness of the content inferred via the idiomatic strings when compared to a literal, less nuanced, paraphrase.

## 5. Testing our presuppositional claims

Given the prominent place that empirical data has played in developing our own hypothesis, it is only appropriate that we employ tests to evaluate our original claims. We go about this in two steps. One is to employ a well-known empirical test that relies on our linguistic intuitions and the other is a more severe experimental pilot that collects psychological measures. This is what we turn to in the next two sections.

### 5.1. Armchair observations

When one hears the utterance *Noemi stopped smoking*, it implies that she smoked in the past (this is *presupposed* content) and that she currently does not smoke (this is often referred to as *at issue* content). One of the standard linguistic-intuition-based tests of presupposition aims to determine whether *presupposed* content projects across a specific range of grammatical contexts, even as these contexts modify the *at issue* content. For example, a presupposition expressed under negation would maintain the existence of presupposed content even as the at-issue content is reversed: Upon hearing *it is not the case that Noemi stopped smoking*, the presupposed content (that she smoked in the past) is maintained, but the at-issue information is reversed, i.e., one would infer that Noemi currently smokes. Thus, the presupposition is said to project. Simons (2005) refers to these projection tests in specific grammatical contexts as Basic Projection Facts, which we list in (7) below (also see Langendoen and Savin, 1971; Chierchia and McConnell-Ginet, 1990):

(7) Given a sentence S which, when uttered, is typically understood to presuppose p,

utterances of a sentence S′ will typically also be understood to presuppose p, where:

a. S′ is the negation of S.

b. S′ is the yes/no question formed from S.

c. S′ is a conditional with S as its antecedent.

d. S′ embeds S under an epistemic modal.

One immediate way to test our idiom-related claims then is to determine whether given presuppositional information that we claim is tied to idioms projects in these grammatical environments in a way similar to the classic presuppositional cases. That is, if the presupposed content appears to remain in the classic test environments, even as the at-issue information might not appear to, this would provide some intuitive support to our claim. Let us consider *Joey broke the ice by making a joke*, which will be adopted later into our experimental task. As part of our armchair test, we transform this phrase and create four new ones that are distributed across the four environments described above. These are expressed as (8a-d):

(8) *Example*: Joey *broke the ice* by making a joke.

a. *Negation*: Joey did not *break the ice* by making a joke.

b. *Question*: Did Joey *break the ice* by making a joke?

c. A*ntecedent of conditional*: If Joey were to *break the ice* by making a joke, it would have no positive effects.

d. *Possibility modal*: Joey might *break the ice* by making a joke.

Again, the question is whether the presupposed content for this particular idiom in this particular sentence – that there was some pre-existing social tension (before an effort was made to relieve that tension) – persists across these grammatical environments. In our reckoning, they do. In (8a), under negation, the asserted content has been negated (the social tension was not relieved or the joke did not succeed) but the presupposed content remains (that there was some pre-existing social tension). In (8b), whether one responds affirmatively or negatively, the felicity of the answer depends on assuming that there was some contextual reason that called on breaking the ice. Likewise for the remaining cases: whether or not the “relieving social tension” meaning is confirmed, reference to presupposed content (reference to the existence of some prior social tension) remains. It appears then that our claim passes its initial test. While we do not want to get ahead of ourselves, it is noteworthy that even if our findings apply to only a subset of idioms, this amounts to a novel characterization of idioms.

### 5.2. Testing our claims experimentally: initial findings

Crucially, by appreciating the presuppositional effects of idiomatic expressions, we can better understand why the use of a given idiom is felicitous only under certain circumstances. To illustrate this, consider the following two examples:

(9) a. Joey was enrolled in a competitive biology course. At the beginning of the semester, no one dared to speak to each other. Therefore, Joey *broke the ice* by making a joke.

b. Joey was enrolled in a competitive biology course. By the end of the semester, everybody in the class got to know each other. Therefore, Joey *broke the ice* by making a joke.

In (9a), *broke the ice* is used felicitously because the array of background assumptions triggered by the idiom (i.e., that there exists social tension among the classmates) is consistent with the assumptions provided by the preceding statement (“no one dared to speak to each other”). In contrast with this, in (9b), the array of background assumptions that the speaker appears to presuppose by the use of *broke the ice* is inconsistent with her preceding statement (“everybody in the class got to know each other”), thus generating the perception of an infelicitous discourse continuation. The contrast between (9a) and (9b) is thus comparable to the contrast discussed in section “Armchair observations” above between cases of satisfied, or contextually supported, presupposition and cases of “presupposition failure” [see our examples in (5a) and (5b)].

In order to more severely test our hypothesis, the first and second authors prepared a pilot experiment (see Griffen and Noveck, 2023) which was based on the insight described above, i.e., that idioms concern not only at-issue information (such as *initiated social contact* for *broke the ice*) but presupposed information as well (*that there was some pre-existing social tension*). In this way, idioms are similar to presuppositions. This led us to develop a paradigm (inspired by a study on presuppositions from Shetreet et al., 2019), in which participants would receive brief vignettes.

In this pilot, 18 idioms were investigated and, for each, a precondition was readily identified. As before, let us provide a couple of other examples. For “bury the hatchet,” which can be loosely translated to mean “make peace,” it presupposes that there was discord previously. For “spill the beans,” which (as described earlier) means something akin to “reveal a secret,” it necessitates that there was a secret to be kept. When such a precondition is satisfied in the discourse, one would expect the idiom’s use to be felicitous and facilitated; when the precondition is not met (or not fully met), it would make the idiom’s use appear infelicitous (or, at least hard to accommodate).

This led us to prepare vignettes for the purposes of a self-paced reading study. Each vignette comes in one of two varieties, one that will support the idiom’s presuppositional content (as in 9a) and one that does not (as in 9b) above (these are combined and reprised together in (10) as we highlight the task’s experimental features). To provide a little variety we also show our vignette for *spill the beans* in (11). Both (10) and (11) underline three experimental features. One is that the slashes indicate the point at which a participant advances the text so there are always five such reading segments. A second is that there were two kinds of second sentences (or second segments), as first shown across (9a) and (9b): one type of second-sentence will later make the idiomatic string felicitous and the other type (in brackets) will make it infelicitous. The third is that the last three segments (the third through fifth) make up the third sentence of the vignette, among which one finds the idiomatic string always occurring in the fourth segment.

(10) Joey was enrolled in a competitive biology course./At the beginning of the semester, no one dared to speak to each other. [By the end of the semester, everybody in the class got to know each other.]/Therefore, Joey/ *broke the ice*/by making a joke.

(11) Nick is organizing a huge birthday party for his mother next week at their house./She knows nothing about the party because it is planned as a surprise [She has taken over the planning because she loves entertaining.]/Last night, Nick/*spilled the beans*/ about the event.

Twenty-four vignettes were presented as part of our self-paced reading task (there were also numerous filler items that had nothing to do with presuppositions or idioms). Eighteen items were devoted to idioms and were distributed randomly across three conditions. Participants received a story context (like the ones in [10] and [11]) that led to an idiom that was (i) felicitous, (ii) infelicitous, or else (iii) a control in which the felicitous context was presented but included an invented nonsense idiom instead. To make this concrete, the control version of (iii) for (11) kept the “felicitous” second sentence above, but replaced *spilled the beans* with *cramped the air*. The 18 idiom-potential vignettes were rotated so that every story context was presented as the source of one of the three conditions and so that every participant received one of the three. All told, an individual participant received six randomly chosen idiomatic strings that were presented in a felicitous context, six whose contexts were infelicitous with respect to the idiom’s presupposed information (and thus required some accommodation), and six that used nonsense idioms (where another idiom would be appropriate). The remaining six items were control items drawn from Shetreet et al.’s EEG study Shetreet et al. (2019) on presuppositions, which recorded reactions to (a specific word in) a sentence that was (a) downstream from a factive presupposition and; (b) that made the sentence either consistent or inconsistent with the prior context. For an example, in the item in (12) below, participants received a second sentence that would make the third sentence appear consistent [or inconsistent] with the previous context:

(12) Bruce taught a class on quantum physics./He **saw** that his students had *mastered* [were *confused* by] the material. /Almost all of them/scored perfectly/on every test.

Items such as these were included as a sanity test. It was assumed that we would extend Shetreet et al. (2019) results (which concerned ERPs to the underlined term in the third sentence) by finding reading time slowdowns for those cases where the third sentence is inconsistent with the presupposition in its context.

Our online participants progressed through each story by pressing the spacebar on their keyboard and would occasionally receive a comprehension question. Importantly, the idiom string, which appeared in the fourth segment, as well as the final segment which appeared in the fifth, ultimately provided dependent variables. Our expectation was that there would be significantly faster reading times for the fifth segment when it appears after a felicitous idiomatic string rather than after an infelicitous one. In other words, we expected slowdowns when the idiomatic string was used in non-felicitous context; likewise, we expected our nonsense idioms to produce slowdowns, too.

To be brief, we can say that our results aligned with our predictions. To provide a little detail, Griffen and Noveck (2023) point out three findings concerning the reading times of the last (the fifth) segment. First of all, the findings extend (Shetreet et al.’s, 2019) outcomes with reading times, which further validates their paradigm and provides a benchmark about the way participants process information that is inconsistent or else consistent with presupposed content. Participants significantly slowed down (by 135 ms) when the fifth segment of the vignette is inconsistent with a prior information carried by the presupposition as opposed to when it is consistent with it. Second, and similarly to the Shetreet et al. cases, for those items in which the second sentence does not provide supporting presuppositional content for the idiom in the third sentence (e.g., when students in the course in item [10] all know each other before *breaking the ice* is used), we also find significant slowdowns compared to cases where the content in the vignette is consistent with the presupposition of the idiom, even though the spread is smaller (slowdowns are about 74 ms). Third, the fifth segments following nonsense idioms prompted by far the slowest reactions (135 ms slower than fifth-segments of vignettes that included conventional idioms but without presuppositional support and 210 ms slower than fifth-segments that included conventional idioms with presuppositional support).

These data are consistent with other recent work (Beck and Weber, 2020) that shows how an idiom, when used in a context that biases a participant toward a “high literality” reading, prompts slowdowns two segments after the idiom. For example, the segment “sooner than later” in (13b) prompts slowdowns compared to cases in which the same segment appears after a figurative meaning is encouraged (13a).

(13) (a) The new schoolboy/ who did not know/anyone in his class/just wanted to/break the ice/with his peers/sooner than later/Monday morning.

(b) The chilly Eskimo/ who was eager/to catch some fish/just wanted to/break the ice/with his peers/sooner than later/Monday morning.

Our presuppositional account would argue that the presuppositional content of *break the ice* is unsatisfied in (13b) but satisfied in (13a).

We note that data from Griffen and Noveck (2023) aim to address cases in which participants receive brief stories. It is not clear how our account can be extended to cases where participants need to make grammatical judgments of idioms versus yoked controls. In our view, the speed advantages for idioms on grammatical judgment tasks might well depend on other features of idioms such as frequency and familiarity (e.g., see Libben and Titone, 2008; Carrol and Conklin, 2020).

## 6. Conclusion

This paper began by describing how idiom processing appears to be exceptional in the context of figurative language literature. The *idiom superiority effect* intriguingly reports faster reading times for idiomatic readings when compared to their literal controls, providing this literature with a unique effect when compared to other investigated figures, such as ironical and metaphorical readings (when compared to their controls). Essentially, we were driven to better understand this paradox. We thus sought to account for the characteristics that idioms tend to hold as we proposed a relevance-theoretic interpretation on their processing. For this reason, we reviewed a relevance-theoretic account offered by Vega-Moreno (2003, 2005), where idioms are treated as conventional metaphors. While we found Vega-Moreno’s approach of treating idioms as conventional metaphors enriching, it did not provide the wherewithal to account for the *idiom superiority effect*.

We subsequently went on to propose that idiomatic strings generate a set of background assumptions, which verge on being presuppositional. This implies that each idiom is considered individually and that each idiomatic string necessitates specific contextual conditions in order to be considered felicitous. It follows that an idiomatic string will appear felicitous if there is contextual support and it will prompt incongruity if there is no contextual support. Our RT-inspired work leads to the conclusion that – if the idiom string is recognized as such and processed as a whole – it will automatically activate some procedure leading to the recovery of its associated background assumptions. Given that these assumptions conflict with the context when this is intended to support a literal interpretation, processing difficulties are to be expected. In other words, according to our approach, idiomatic interpretations will tend to be produced even if they are being used in literal control conditions. In this way, the *idiom superiority effect* is a natural consequence of our analysis. Of the three prominent idiom processing accounts in the psycholinguistic literature (as described in the Introduction) – the *compositional, direct retrieval* and *hybrid* accounts – our proposal is most compatible with the last two because we would have to assume that idiomatic meanings are generated (at some point) even in the literal control conditions, thus producing incongruities and slowdowns.

Turning to the RT literature, our explanation of idioms and their role in the *idiom superiority effect* is actually consistent with prior analyses of some key properties of procedural meaning. Specifically, Escandell-Vidal and Leonetti (2011) have suggested that procedural meaning is characterized by so-called ‘rigidity’, so that “procedural meaning will always prevail (i.e., impose its conditions) even when it enters into contradiction with other kinds of information, both linguistically encoded and contextually inferred” (2011, p. 81). Based on this, they maintain that for interpretation to succeed, the instructions encoded by an item must be satisfied and, as a consequence, any possible mismatch must be resolved by preserving the representations obtained by following the instructions. This rigidity is evident in the many cases of idiomatic expression that we discussed and that lead to mismatching contexts. In the example *Has the love of my life, my inspiration, Tom, kicked the bucket yet*? (which is a modification of [6]), it is interesting to notice that, as the range of assumptions and attitudinal disposition recovered by following the procedure encoded by the idiom cannot be overridden by the conceptual information encoded by the expressions *love of my life* and *my inspiration*; the only possible way to achieve a sensible and relevant interpretation of the utterance is to adopt an ironical interpretation of these expressions. Such an ironical interpretation would preserve the background assumptions derived by the application of a rigid procedure and resolve the mismatch at issue.

In sum, through the examples that we have provided and the preliminary results from both linguistic-intuition-based tests and ongoing experimental work, we hope that this paper can serve as an introductory, albeit convincing, argument for viewing idioms as a class of unique figurative expressions with their own processing requirements. One of these, which has been largely overlooked, is the way idioms include a procedural meaning that takes into account relevant presuppositional information. Once this feature of idioms is taken into consideration, one is in a better position to account for the *idiom superiority effect*. As we have outlined in this paper, our next step will be to follow up on our pilot experiment. It is our hope that our novel approach to idiom comprehension will enrich discussions of figurative language and its processing.

## Data availability statement

Further inquiries about original findings can be directed to the second author or the corresponding author.

## Author contributions

The authors made the following contributions. IN: conceptualization, writing – original draft preparation, writing – review and editing, experiment conceptualization and preparation, project administration, supervision, and methodology. NG: conceptualization, writing – draft preparation, editing, experimental preparation, and data analysis. DM: conceptualization, writing – original draft preparation, writing – review and editing, and relevance theoretic analyses. All authors contributed to the article and approved the submitted version.

## Funding

This work was supported by seed money from the Laboratoire d’excellence Empirical Foundations of Linguistics (Labex-EFL) in Paris as well as from a Chaire d’Excellence grant from the Université de Paris-Cité to IN, a doctoral grant from the Université de Paris to NG, and an SNSF Eccellenza Grant (PCEGP1_186931/1) to DM.

## Conflict of interest

The authors declare that the research was conducted in the absence of any commercial or financial relationships that could be construed as a potential conflict of interest.

## Publisher’s note

All claims expressed in this article are solely those of the authors and do not necessarily represent those of their affiliated organizations, or those of the publisher, the editors and the reviewers. Any product that may be evaluated in this article, or claim that may be made by its manufacturer, is not guaranteed or endorsed by the publisher.

## Footnotes

^1^There are other hybrid accounts and there appears to be disagreement about the way to classify the Configuration Hypothesis (e.g., see Titone et al., 2019). We are not concerned with these distinctions here because our goal is to provide background concerning psycholinguistic accounts on idioms before considering approaches inspired by Relevance Theory later.

^2^There are multiple post-Gricean approaches, such as the *Constraint Satisfaction* view (Pexman et al., 2000; Pexman, 2008), which states that multiple factors can influence processing of irony in parallel, including the speaker’s reputation and role, that can result in equivalent speeds for comprehending ironic sentences and their literal counterparts (Ivanko and Pexman, 2003; Katz et al., 2004). We do not aim to summarize all current accounts of figurative processing here, but to provide the academic experimental context in which Relevance Theory operated before turning to our approach to idiom processing.
